# Therapeutic potential for phenytoin: targeting Na_v_1.5 sodium channels to reduce migration and invasion in metastatic breast cancer

**DOI:** 10.1007/s10549-012-2102-9

**Published:** 2012-06-08

**Authors:** Ming Yang, David J. Kozminski, Lindsey A. Wold, Rohan Modak, Jeffrey D. Calhoun, Lori L. Isom, William J. Brackenbury

**Affiliations:** 1 Department of Biology, University of York, Heslington, York, YO10 5DD UK; 2 Department of Pharmacology, University of Michigan Medical School, Ann Arbor, MI 48109-5632 USA

**Keywords:** Electrophysiology, Invasion, Metastasis, Migration, Phenytoin, Voltage-gated Na^+^ channel

## Abstract

Voltage-gated Na^+^ channels (VGSCs) are heteromeric membrane protein complexes containing pore-forming α subunits and smaller, non-pore-forming β subunits. VGSCs are classically expressed in excitable cells, including neurons and muscle cells, where they mediate action potential firing, neurite outgrowth, pathfinding, and migration. VGSCs are also expressed in metastatic cells from a number of cancers. The Na_v_1.5 α subunit (encoded by *SCN5A*) is expressed in breast cancer (BCa) cell lines, where it enhances migration and invasion. We studied the expression of *SCN5A* in BCa array data, and tested the effect of the VGSC-blocking anticonvulsant phenytoin (5,5-diphenylhydantoin) on Na^+^ current, migration, and invasion in BCa cells. *SCN5A* was up-regulated in BCa samples in several datasets, and was more highly expressed in samples from patients who had a recurrence, metastasis, or died within 5 years. *SCN5A* was also overexpressed as an outlier in a subset of samples, and associated with increased odds of developing metastasis. Phenytoin inhibited transient and persistent Na^+^ current recorded from strongly metastatic MDA-MB-231 cells, and this effect was more potent at depolarized holding voltages. It may thus be an effective VGSC-blocking drug in cancer cells, which typically have depolarized membrane potentials. At a concentration within the therapeutic range used to treat epilepsy, phenytoin significantly inhibited the migration and invasion of MDA-MB-231 cells, but had no effect on weakly metastatic MCF-7 cells, which do not express Na^+^ currents. We conclude that phenytoin suppresses Na^+^ current in VGSC-expressing metastatic BCa cells, thus inhibiting VGSC-dependent migration and invasion. Together, our data support the hypothesis that *SCN5A* is up-regulated in BCa, favoring an invasive/metastatic phenotype. We therefore propose that repurposing existing VGSC-blocking therapeutic drugs should be further investigated as a potential new strategy to improve patient outcomes in metastatic BCa.

## Introduction

Breast cancer (BCa) is the most common cancer in women, and the leading cause of female cancer-related deaths worldwide [1]. Metastasis is the main cause of mortality and is therefore a critical therapeutic target [2]. Treatment options currently available to patients with metastatic BCa are largely limited to palliation [3]. Thus, there is an urgent need to identify new molecular targets and adjuvant therapies with curative intent.

Voltage-gated Na^+^ channels (VGSCs) are heteromeric membrane protein complexes composed of one pore-forming α subunit and smaller β subunits [4]. Inward flow of Na^+^ through VGSCs is responsible for the depolarizing phase of action potentials in neurons and muscle cells [5]. There are nine α subunits (Na_v_1.1–Na_v_1.9) and four β subunits (β1–β4) [4]. The β subunits contain an extracellular immunoglobulin loop [6]. While they do not form the ion conducting pore, they modulate channel gating, and are members of the immunoglobulin superfamily of cell adhesion molecules (CAMs) [7]. VGSCs play a key role in organogenesis of the developing central nervous system [8]. VGSC α and β subunits function within complexes in neurons to regulate electrical excitability, neurite outgrowth, pathfinding, and migration [9–11].

VGSCs are widely expressed in metastatic cells from a number of cancers, including BCa (reviewed in [8]). For example, *SCN5A* (encoding Na_v_1.5), *SCN8A* (encoding Na_v_1.6), and *SCN9A* (encoding Na_v_1.7) mRNAs have been detected in BCa cell lines [12]. Of these, a neonatal splice variant of *SCN5A* is most abundant, and its mRNA is ~1,800-fold higher in strongly metastatic MDA-MB-231 cells than weakly metastatic MCF-7 cells [12]. Na^+^ currents have been recorded in MDA-MB-231 cells, but are absent in weakly metastatic MCF-7 cells [12, 13]. Neonatal *SCN5A* mRNA expression in BCa biopsies correlates with occurrence of lymph node metastasis [12]. Suppression of Na_v_1.5 in MDA-MB-231 cells, either with the pore-blocking tetrodotoxin (TTX), function-blocking antibodies, or with siRNA, inhibits cellular behaviors associated with metastasis, including detachment, migration, galvanotaxis, and invasion [12–15]. Na^+^ current carried by Na_v_1.5 enhances the cells’ invasiveness by promoting cysteine cathepsin activity in caveolae [16, 17]. In contrast to Na_v_1.5, the VGSC β1 subunit functions as a CAM in BCa cells, enhancing adhesion [18]. Thus, VGSC α and β subunits appear to play dynamic roles in regulating cell adhesion, migration, and invasion in BCa.

Phenytoin (5,5-diphenylhydantoin), a class 1b antiarrhythmic agent and widely used antiepileptic drug, is a potent blocker of VGSCs (IC_50_ ~10 μM) [19, 20]. It also inhibits delayed rectifier human *Ether*-*à*-*go*-*go*-related gene (HERG) K^+^ channels at significantly higher concentrations (IC_50_ > 300 μM) [21]. The affinity of VGSCs for phenytoin is increased when they are in their inactivated state, following sustained membrane depolarization or high frequency channel activation, e.g., during action potential firing in neurons [20]. Phenytoin inhibits prostate-specific antigen (PSA) and interleukin-6 (IL-6) secretion, and migration in prostate cancer cells [22, 23]. It also suppresses endocytosis in small cell lung cancer cells [20, 24]. However, the effect of phenytoin on VGSC currents and metastatic cell behavior in BCa cells is unknown.

Our aims here were to (1) study the expression of *SCN5A* in published BCa array data and (2) assess the effect of phenytoin on Na^+^ current, migration, and invasion in BCa cells. We demonstrate that *SCN5A* is up-regulated in BCa samples in several datasets, and associates with poor prognosis. In addition, phenytoin inhibits Na^+^ current, migration, and invasion in metastatic BCa cells in vitro. We propose that VGSCs may be a promising target for therapeutic intervention in BCa using existing VGSC-inhibiting drugs. Furthermore, phenytoin, as a widely used FDA-approved oral anticonvulsant, should be further studied as a potential, cost-effective, new treatment approach.

## Methods

### *In silico* analysis

*SCN5A* expression in BCa microarrays was studied using the web-based Oncomine database, as described previously [25–27]. Normalization and statistical analysis were performed in Oncomine using the standard settings: for each array, data were log_2_-transformed, median centered, and standard deviation normalized to one [25]. Fold changes <1.3-fold were not considered significant because such small changes are often not reproducible by quantitative PCR validation [28–30]. Cancer outlier profile analysis (COPA) was used to evaluate *SCN5A* outlier expression in a subset of BCa samples [31]. Outlier expression was defined as being in the top 10 % of COPA scores at any of three percentile cutoffs (75th, 90th, and 95th). Where applicable, REMARK reporting criteria have been used [32]. Patients, specimen characteristics and assay methods are detailed in the reference cited for each dataset, and at www.oncomine.org.

### Cell culture

MCF-7 and MDA-MB-231 cells were grown in Dulbecco’s modified eagle medium supplemented with 5 % fetal bovine serum and 4 mM l-glutamine [12]. Cells were confirmed to be mycoplasma-free by 4′,6-diamidino-2-phenylindole (DAPI) method [33]. Molecular identity was confirmed by short tandem repeat analysis [34].

### Immunocytochemistry, confocal microscopy, and image analysis

Immunocytochemistry and confocal microscopy were performed as in Refs. [9, 10]. Samples were labeled with a monoclonal pan-VGSC α subunit antibody (1:100; Sigma), polyclonal anti-β1 antibody (1:2,000) [35] or polyclonal anti-GM130 antibody (1:1,500; Proteintech), Alexa Fluor-conjugated phalloidin (1:40; Molecular Probes), and DAPI. Images were processed and analyzed using ImageJ software (NIH). The intensity profiles of VGSC α subunit and phalloidin were determined using the “straight line profile” function drawn across lamellipodia into the cell body, as in Refs. [36, 37]. For both channels, peak signal intensity in lamellipodia (defined as the peak in phalloidin labeling) was expressed as a ratio of the mean signal intensity 5–10 μm inside the plasma membrane. Measurements (3 per cell) were taken from ≥12 cells per line.

### Electrophysiology

The whole-cell patch clamp technique was used to record membrane Na^+^ currents from cells grown on glass coverslips [18]. Voltage-clamp recordings were made using a Multiclamp 700B amplifier (Molecular Devices) compensating for series resistance by 40–60 %. Currents were digitized using a Digidata 1440A interface (Molecular Devices), low-pass filtered at 10 kHz, sampled at 50 kHz, and analyzed using pCLAMP 10.3 software (Molecular Devices). Linear components of leak were subtracted using a P/6 protocol [38]. Data manipulation and curve fitting were performed as before [9].

### Pharmacology

Phenytoin sodium salt (Sigma) was prepared as a 180 mM stock dissolved in 75 mM NaOH. It was frozen in aliquots, then thawed and diluted in culture medium to 5–200 μM, as required. Control cells were treated with the final working concentration of NaOH (2–83 μM). In assays that exceeded 24 h, treatments were replaced daily.

### Viability

The cytotoxicity of phenytoin was determined using a trypan blue exclusion assay [39]. Cells (5 × 10^4^) were plated in 35 mm dishes. The next day, dishes were treated each with phenytoin or vehicle. After 24 h, the number of live versus dead cells was determined from 20 fields of view per dish. Results were compiled from three experimental repeats.

### Proliferation

Cells (3 × 10^4^ per well) were seeded in 12-well plates. The following day, triplicate wells were treated each with phenytoin or vehicle for 24 h. The number of cells per well was determined using the colorimetric 3-[4,5-dimethylthiazol-2-yl]-2,5-diphenyltetrazolium bromide (MTT) assay [40]. Results were compiled as the mean of three repeats.

### Motility

Cellular motility was determined using a wound healing assay, as described previously [23]. Cells (2 × 10^5^) were seeded in 35 mm dishes. The following day, three wounds were made per dish using a P1000 pipette tip. Dishes were rinsed once in fresh medium, and wound widths were immediately measured (*W*_0_) using an inverted microscope with graticule at 45 fixed points per dish (pre-labeled on the underside of the dish with a pen). Dishes were then treated with phenytoin or vehicle for 24 h and the same sites were subsequently re-measured (*W*_t_). For each site, a migration index (MI) was calculated as MI = 1 − (*W*_t_/*W*_0_). Means were compiled from three repeat experiments, giving at least 135 data points for analysis.

### Invasion

Cell culture inserts for 24-well plates, with 8 μm pores, were coated with extracellular matrix gel (Sigma). Cells (5 × 10^4^/ml) were plated in triplicate in a 0.1–1 % fetal bovine serum chemotactic gradient and incubated with phenytoin (50 μM) or vehicle for 48 h. The number of invaded cells was determined using the MTT assay [12, 40]. Results were compiled as the mean of three repeats.

### Data analysis

Data are presented as mean and SEM unless stated otherwise. Statistical analysis was performed using GraphPad Prism 5.0d. Normal distribution was determined using D’Agostino–Pearson omnibus test. Pairwise statistical significance was determined with *t* tests, or Mann–Whitney tests. Multiple comparisons were made using ANOVA and Tukey post hoc tests, or Kruskal–Wallis with Dunns tests, as appropriate*. P* values computed by Oncomine were corrected for multiple comparisons by Bonferroni method [25]. Predictive value of *SCN5A* was assessed using receiver operating characteristic (ROC) curves. Kaplan–Meier curves for overall survival were compared by log-rank tests. Percent survival and hazard ratios are presented with 95 % confidence intervals. Results were considered significant at *P* < 0.05 (*).

## Results

### *SCN5A* is expressed in patient BCa samples and is predictive of poor prognosis

**Fig. 1:**
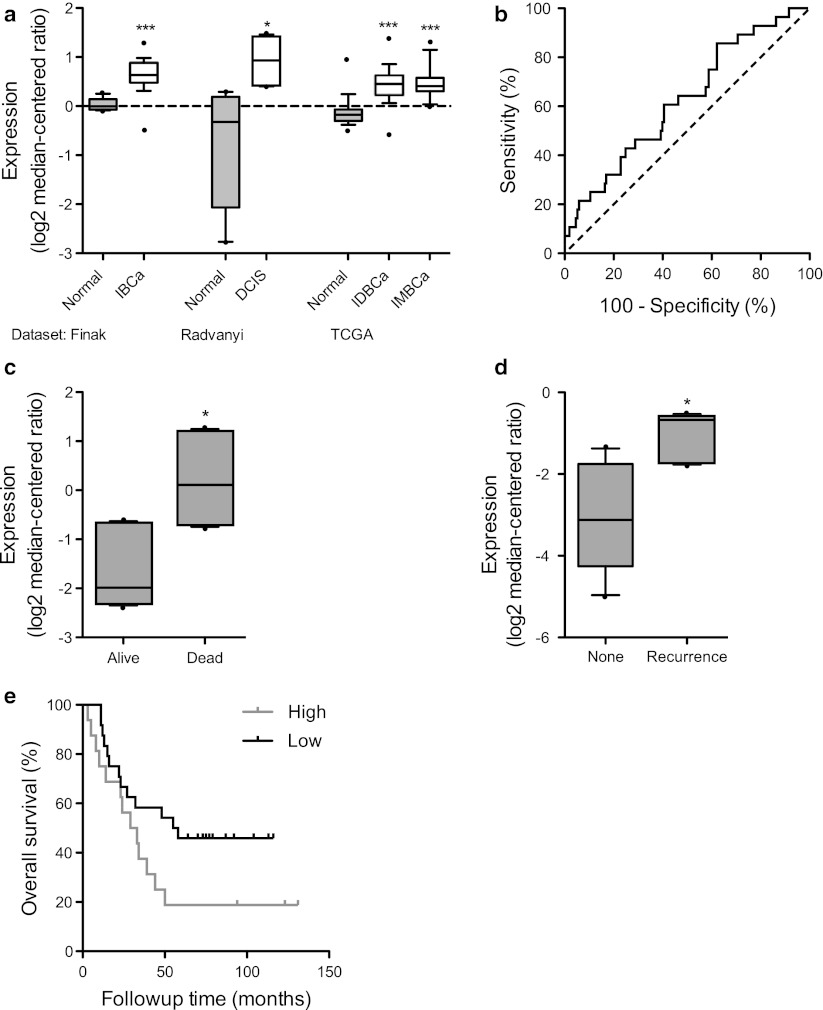
*SCN5A* is up-regulated in breast tumors and associates with poor prognosis. **a** Expression of *SCN5A* in invasive breast cancer (IBCa), ductal carcinoma in situ (DCIS), invasive ductal breast carcinoma (IDBCa), or invasive mixed breast carcinoma (IMBCa), versus normal breast in three datasets analyzed in Oncomine: [41] (*n* = 59); [42] (*n* = 7); and The Cancer Genome Atlas (TCGA; *n* = 371). **b** Receiver operating characteristic (ROC) curve analysis of prediction of metastasis at five years in [43] (*n* = 181). **c** Comparison of *SCN5A* expression between those with/without recurrence at five years in [44] (*n* = 8). **d** Comparison of *SCN5A* expression between patients with invasive breast carcinoma alive or dead at five years in TCGA dataset (*n* = 6). **e** Kaplan–Meier survival analysis comparing overall survival of those with high versus low *SCN5A* expression in Ref. [45] (*n* = 40). *Box plot dots* maximum and minimum values; *whiskers* 90th and 10th percentile values; and *horizontal lines* 75th, 50th, and 25th percentile values. *P < 0.05; ***P < 0.001

Na_v_1.5 (in its neonatal splice form) is present in BCa biopsies, correlating with lymph node metastasis [12]. Na_v_1.5 is also expressed in MDA-MB-231 cells, where it potentiates invasion and migration [12, 13, 15]. In order to extend these observations to clinically relevant datasets, we used the Oncomine database to compare the expression of *SCN5A* in normal breast and BCa samples across multiple microarrays [25]. *SCN5A* was expressed at significantly higher levels in BCa (including ductal carcinoma in situ, and invasive, ductal and metastatic BCa), compared with normal breast tissue, in three out of seven datasets for which differential data were available [1.5-fold, *P* < 0.001, [41] and The Cancer Genome Atlas (TCGA) dataset^1^; and 3.6-fold, *P* < 0.05 [42] (Fig. 1a)]. There was no relationship between *SCN5A* expression and estrogen receptor (ER), progesterone receptor (PR), or human epidermal growth factor 2 (HER2) status (Table 1).

**Table 1:** Relationship between ER/PR/HER2 status and *SCN5A* expression

Relationship	Fold change in *SCN5A* expression	P	Dataset
ER^+^ versus ER^−^	0.93	0.60	TCGA
ER^+^ versus ER^−^	1.21	0.97	[41]
PR^+^ versus PR^−^	0.99	0.80	TCGA
PR^+^ versus PR^−^	1.08	0.95	[41]
HER2^+^ versus HER2^−^	1.18	0.06	TCGA
HER2^+^ versus HER2^−^	0.83	0.82	[41]
Triple negative versus other	1.04	0.73	TCGA
Triple negative versus other	1.21	0.95	[41]

*ER* estrogen receptor, *PR* progesterone receptor, *HER2* human epidermal growth factor 2, *TCGA* The Cancer Genome Atlas

Data are shown for datasets in which *SCN5A* is elevated in BCa samples ([41] and TCGA dataset). ER/PR/HER2 status is not available for [42]

We next studied the prognostic value of *SCN5A* expression. *SCN5A* was more highly expressed in tumor samples from patients who subsequently developed metastases than from those who did not within 1 year (*P* < 0.05), 3 years (*P* < 0.01), and 5 years (*P* < 0.01) [43]. However, the up-regulation of *SCN5A* (1.3-fold) was at the limit of significance [28–30]. Nonetheless, ROC analysis revealed that *SCN5A* expression was effective at predicting metastasis [area under the curve (AUC) = 0.63 ± 0.06; *P* < 0.05; Fig. 1b]. *SCN5A* was more highly expressed in those who experienced recurrence within 5 years than from those who did not (4.1-fold; *P* < 0.05; Fig. 1c [44]). In addition, *SCN5A* expression was higher in patients who were dead at 5 years (3.6-fold, *P* < 0.05, Fig. 1d; TCGA dataset). High *SCN5A* expression (cut-off at 60th percentile) associated with reduced survival (overall survival at 60 months: 45.4 % [25.3–63.5] for “low” *SCN5A* expression, and 18.5 % [4.3–40.0] for “high” *SCN5A* expression; hazard ratio = 2.1 [0.93–4.84]), although this was not quite statistically significant (*P* = 0.07; log-rank test; Fig. 1e [45]).

**Fig. 2:**
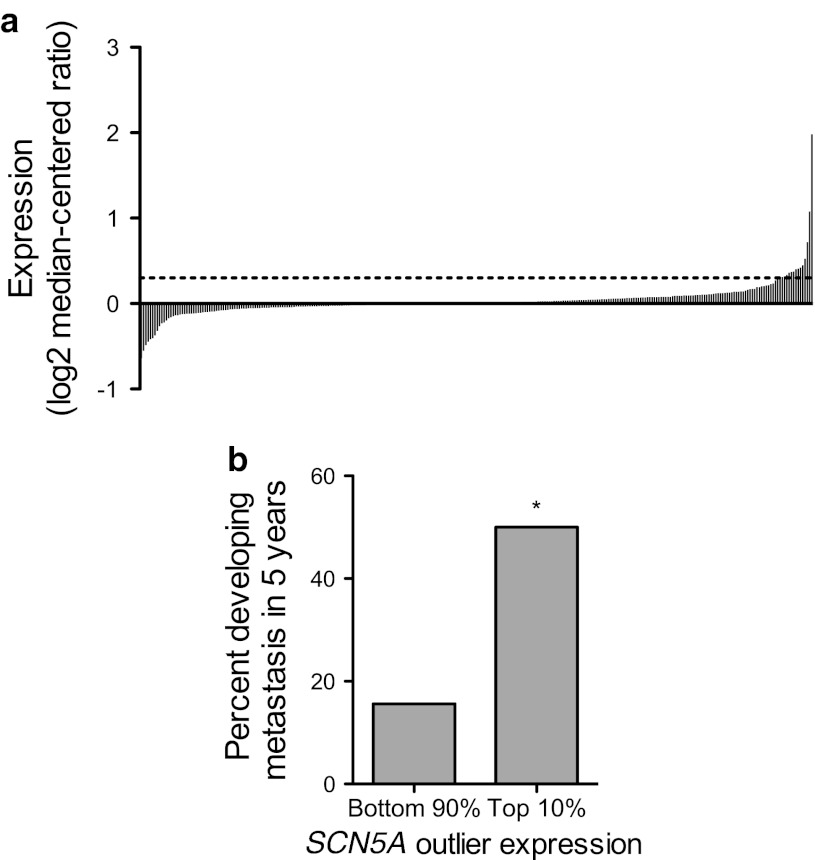
*SCN5A* expression as an outlier associates with metastasis. **a** *SCN5A* (normalized expression units) is shown for all profiled samples in [70]. *Horizontal line* 95th percentile cut-off, above which extend the top 5th percentile samples. **b** Percentage of those that developed metastasis within five years is shown for the 10 % most highly expressing, and *bottom* 90 % of samples in [43]. *P < 0.05 (*n* = 181)

We next used COPA in Oncomine to investigate whether *SCN5A*, similar to other heterogeneously activated oncogenes [31], was expressed as an outlier. An outlier profile occurs when a gene is highly expressed in a fraction of samples in the total population. *SCN5A* was overexpressed in the top 10 % of outliers at the 75th, 90th, and 95th percentiles, across five datasets (mean COPA score: 12.4 ± 4.3; Fig. 2a; Table 2). *SCN5A* overexpression as an outlier (cut-off at 90th percentile) in the primary tumor was associated with developing metastasis within 5 years [odds ratio = 3.2 (1.1–9.4); *P* < 0.05; Fig. 2b) [43]. In summary, *SCN5A* expression is higher in BCa than normal breast across several datasets in Oncomine, and is higher in BCa samples from patients who developed metastasis, recurrence, or who died within 5 years. Furthermore, *SCN5A* is overexpressed as an outlier in a subset of samples, and associates with increased odds of developing metastasis.

**Table 2:** Cancer outlier profile analysis (COPA) of *SCN5A* expression in BCa

Gene rank	Percentile	COPA score	Dataset
93 (in top 2 %)	75	23.8	[69]
223 (in top 2 %)	95	5.8	[70]
440 (in top 9 %)	95	43.2	[69]
483 (in top 10 %)	90	31.6	[69]
657 (in top 8 %)	95	3.6	[71]
791 (in top 6 %)	75	1.3	[70]
792 (in top 5 %)	90	3.9	[72]
1011 (in top 7 %)	95	5.2	[72]
1065 (in top 6 %)	75	2.7	[73]
1112 (in top 7 %)	95	12.7	[73]
1286 (in top 9 %)	90	2.9	[70]

*SCN5A* expression is ranked against other outliers in the dataset at the given percentile (75th, 90th, 95th), according to COPA score. Higher rank and COPA score indicate a more significant outlier profile [31]

### VGSC α and β subunits are expressed in BCa cell lines

**Fig. 3:**
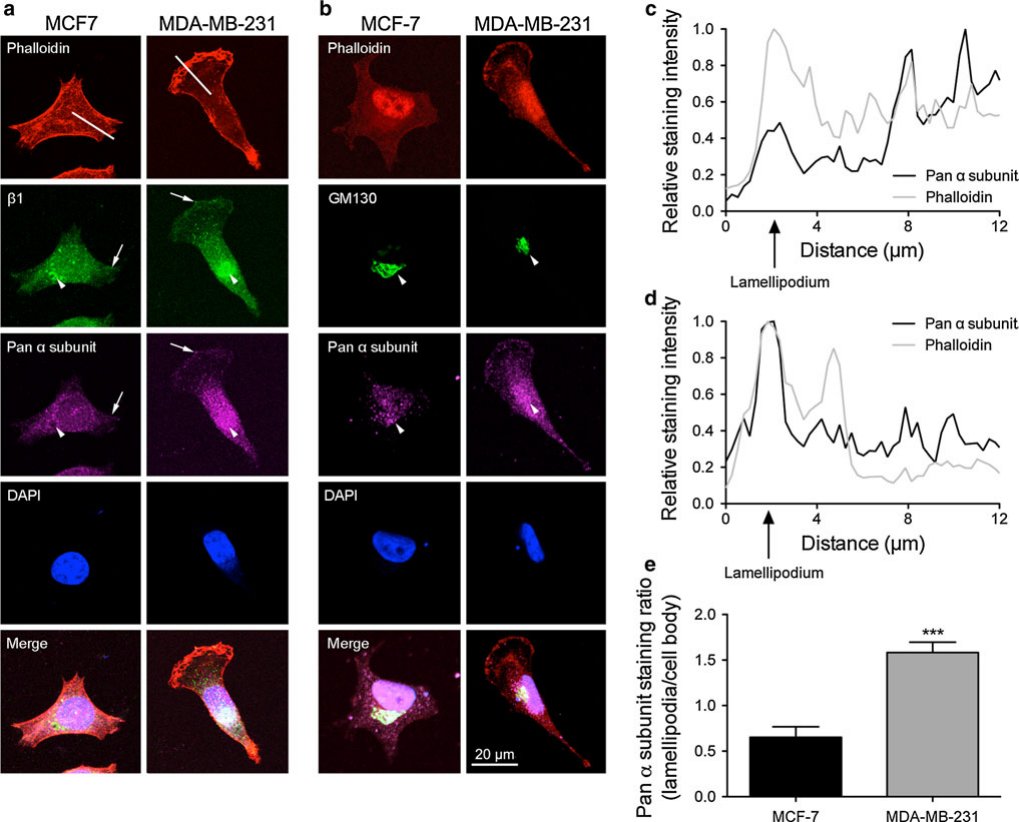
Subcellular distribution of voltage-gated Na^+^ channel α and β1 subunits. **a** MCF-7 and MDA-MB-231 cells labeled with pan-VGSC α subunit and β1 antibodies (*magenta and green*, respectively), phalloidin to label actin cytoskeleton (*red*), and DAPI to label nucleus (*blue*). **b** MCF-7 and MDA-MB-231 cells labeled with pan-VGSC α subunit antibody (*magenta*) and GM130 antibody (Golgi marker; *green*), phalloidin (*red*), and DAPI (*blue*). *Arrows* indicate co-expression of α and β1 at the cell edge. *Arrowheads* indicate perinuclear expression of α and β1, colocalizing with GM130 (**b**). Intensity profiles (normalized to maximum signal) for pan-VGSC α subunit and phalloidin across representative lamellipodia indicated by *lines* in (**a**) are shown for MCF-7 (**c**) and MDA-MB-231 (**d**) cells. **e** VGSC α subunit intensity in lamellipodia relative to internal signal, for MCF-7 and MDA-MB-231 cells. Data are mean ± SEM (*n* ≥ 36). ***P < 0.001

Strongly metastatic MDA-MB-231 cells express significantly more neonatal Na_v_1.5 protein than weakly metastatic MCF-7 cells [12]. By contrast, β1 is more highly expressed in MCF-7 cells than MDA-MB-231 cells [18]. Here, we studied the subcellular distribution of VGSC α and β1 subunits in MDA-MB-231 and MCF-7 cells by confocal immunocytochemistry. Given that β1 can modulate Na^+^ current carried by Na_v_1.5 [18], and that β1-mediated process extension in neurons requires Na^+^ current [9], we hypothesized that α and β1 subunits colocalize at the plasma membrane of BCa cells, as in neurons [9]. We used a pan-specific α subunit antibody, which will detect not only Na_v_1.5 but also Na_v_1.6 and Na_v_1.7, which have also been detected in these cell lines at mRNA level [12]. We found that in both MCF-7 and MDA-MB-231 cells, α subunits were expressed throughout the cytoplasm, and on perinuclear internal membranes, colocalizing with β1 and the Golgi marker GM130 (Fig. 3a, b, arrowheads). This pattern of expression is consistent with previous reports from us and other groups showing perinuclear VGSC expression inside neurons, HEK-293, and cancer cells [15, 17, 36, 46, 47]. Importantly, α and β1 were also colocalized along lamellipodia, defined by phalloidin labeling of F-actin (Fig. 3a, arrows). Line profiles drawn across lamellipodia revealed that α subunits were highly expressed at the lamellipodial plasma membrane of MDA-MB-231 cells, colocalizing with a peak in phalloidin staining, but less so in MCF-7 cells (Fig. 3c, d). The ratio of lamellipodial/cell body staining was significantly higher in MDA-MB-231 than MCF-7 cells (*P* < 0.001; Fig. 3e). This suggests that α subunits are more highly expressed at the lamellipodia of MDA-MB-231 cells than MCF-7 cells. Given that the neonatal Na_v_1.5 splice variant is more highly expressed in MDA-MB-231 cells than MCF-7 cells [12], the α subunit immunoreactivity in MCF-7 cells may represent other variant(s) of Na_v_1.5 that have impaired conduction [48], or other subtypes, e.g., Na_v_1.6 or Na_v_1.7 [12]. In summary, the arrangement of α subunits and β1 at lamellipodia is consistent with their functioning within complexes in these regions to regulate adhesion and migration.

### Phenytoin inhibits Na^+^ currents in MDA-MB-231 cells

**Fig. 4:**
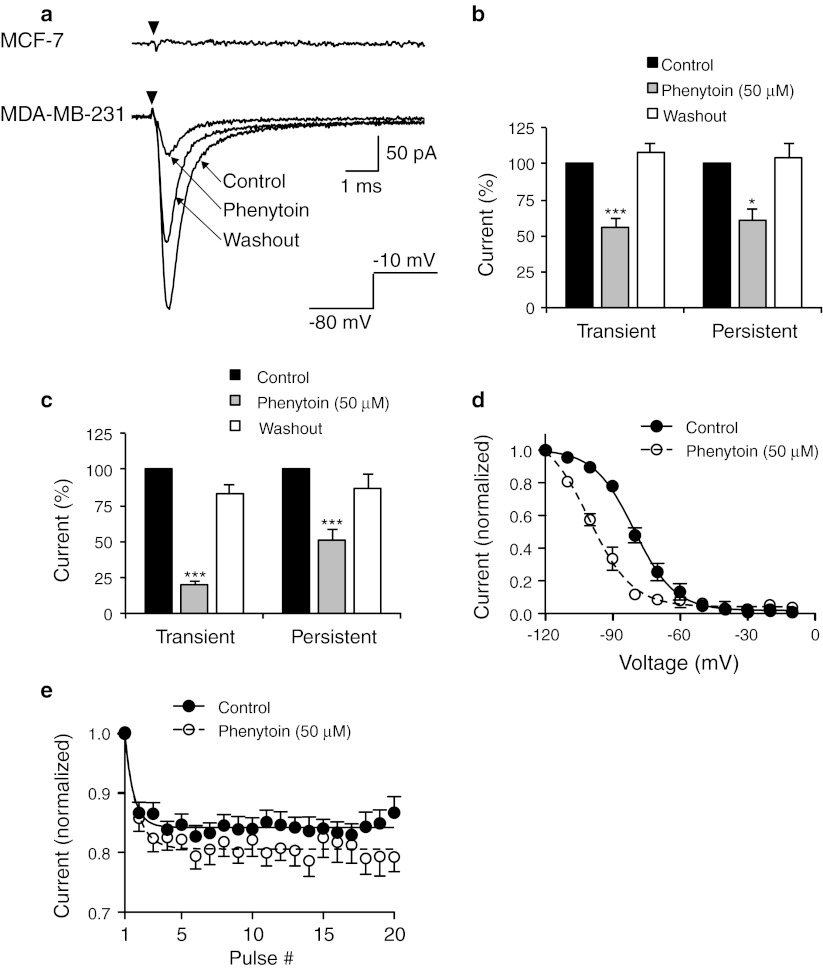
Effects of phenytoin on Na^+^ current. **a** Typical whole-cell recordings from MCF-7 cell (*top*) and MDA-MB-231 cell (*bottom*) following depolarization to −10 mV (*black arrows*) from a holding potential of −80 mV. Na^+^ current in MDA-MB-231 cell is shown in control solution, following perfusion with 50 μM phenytoin, and drug washout. **b** Tonic block (%) of transient and persistent current in MDA-MB-231 cells (activated by depolarization to −10 mV from a holding potential of −120 mV) following perfusion with 50 μM phenytoin, and drug washout. **c** Tonic block (%) of transient and persistent current in MDA-MB-231 cells (activated by depolarization to −10 mV from a holding potential of −80 mV) following perfusion with 50 μM phenytoin, and drug washout. **d** Steady-state inactivation in MDA-MB-231 cells. Normalized current, elicited by 60 ms test pulses at −10 mV following 250 ms conditioning pulses between −120 and −10 mV, applied from a holding potential of −80 mV, plotted as a function of the prepulse voltage for cells in control and following perfusion with 50 μM phenytoin. Data are fit with Boltzmann functions. **e** Use-dependent block of transient current in MDA-MB-231 cells, elicited by 50 Hz pulse trains to 0 mV, applied from a holding potential of −120 mV, normalized to the current evoked by the first pulse plotted as a function of the pulse number for cells in control and following perfusion with 50 μM phenytoin. Data are fit with single exponential functions, which are significantly different between control and phenytoin (*P* < 0.001). Data are mean ± SEM (*n* ≥ 7). *P < 0.05; ***P < 0.001

In order to explore the therapeutic potential of Na_v_1.5 expression in BCa, we next tested the effect of a widely used VGSC-blocking anticonvulsant drug, phenytoin (50 μM), on Na^+^ current in BCa cells, using whole-cell patch clamp recording. This concentration is within the serum therapeutic range used in clinical settings for treatment of epilepsy (10–20 μg/ml) [49]. The inhibition of neuronal VGSCs, e.g., Na_v_1.2, by phenytoin is well established [20]. However, the effect of phenytoin on Na_v_1.5-mediated Na^+^ current in BCa cells has not been investigated. Consistent with previous reports [12, 13], we did not detect any voltage-activated Na^+^ currents in weakly metastatic MCF-7 cells (*n* = 10 cells recorded; Fig. 4a, upper trace). We therefore focused our electrophysiological analysis on MDA-MB-231 cells, which express robust Na^+^ currents [15].

We first studied the tonic block of Na^+^ current, which arises from low-affinity binding of phenytoin to VGSCs in their resting state. Following step depolarization of MDA-MB-231 cells to −10 mV, the VGSCs opened, resulting in a “transient” inward Na^+^ current that decayed toward baseline within a few milliseconds due to VGSCs rapidly entering the inactivated state (Fig. 4a, lower trace). The VGSC inactivation was incomplete, and a small steady-state “persistent” Na^+^ current (approximately 5 % of the transient peak) continued to flow until the end of the depolarizing step (Fig. 4a). Perfusion of phenytoin (50 μM) onto cells during the recording significantly and reversibly reduced the amplitude of the transient and persistent Na^+^ currents (Fig. 4a). When cells were depolarized to −10 mV from a holding potential of −120 mV, the transient current was inhibited by 43.3 ± 5.4 % (*P* < 0.001; Fig. 4b; Table 3). Similarly, the persistent current (measured as mean inward current between 45 and 50 ms following depolarization) was inhibited by 42.4 ± 8.0 % (*P* < 0.05; Fig. 4b; Table 3). The tonic block following depolarization to −10 mV from a less negative holding potential of −80 mV was considerably larger: transient current was inhibited by 79.9 ± 2.2 %, and persistent current was inhibited by 49.1 ± 7.4 % (*P* < 0.001; Fig. 4c; Table 3). Phenytoin also caused a hyperpolarizing shift in the voltage-dependence of steady-state inactivation, shifting the voltage at which half the channels were inactivated (*V*_1/2_) from −79.0 ± 2.0 to −104.4 ± 4.8 mV (*P* < 0.001; Fig. 4d; Table 3).

**Table 3:** Effect of phenytoin on Na^+^ current parameters in MDA-MB-231 cells

Parameter	Control	Phenytoin (50 μM)
Tonic block, *V*_m_ = −120 mV		
Transient (%)	–	43.3 ± 5.4
Persistent (%)	–	42.4 ± 8.0
Tonic block, *V*_m_ = −80 mV		
Transient (%)	–	79.9 ± 2.2
Persistent (%)	–	49.1 ± 7.4
Inactivation *V*_1/2_ (mV)	−79.0 ± 2.0	−104.4 ± 4.8
Inactivation *k* (mV)	−8.3 ± 1.0	−10.9 ± 1.9

*V*_m_ membrane potential, *V*_1/2_ half inactivation voltage, *k* slope factor

We next studied the use-dependent block of Na^+^ current by phenytoin. Repeated depolarization from −120 to 0 mV, at a frequency of 50 Hz, caused a rapid decline in current amplitude that reached a plateau of 84.2 % of initial current after the fourth pulse (Fig. 4e). In the presence of phenytoin, the decline in current reached a plateau of 80.6 % after the fifth pulse (Fig. 4e). Thus, phenytoin caused a small increase in the use-dependent rundown of Na^+^ current in MDA-MB-231 cells.

Phenytoin caused a tonic inhibition of transient and persistent Na^+^ current in MDA-MB-231 cells, which was larger at more depolarized holding potentials. This dependence of tonic block on holding potential has been reported previously, e.g., [20, 50], and is due to phenytoin having a higher affinity for channels in the inactivated than the resting state. Consistent with previous reports, the resting membrane potential of MDA-MB-231 cells was −20.3 ± 0.9 mV (*n* = 5) [12]. At this voltage, the majority of VGSCs are likely to be in the inactivated state, thus phenytoin would likely be a highly potent blocker of the remaining persistent Na^+^ current.

### Phenytoin inhibits migration and invasion of BCa cells

**Fig. 5:**
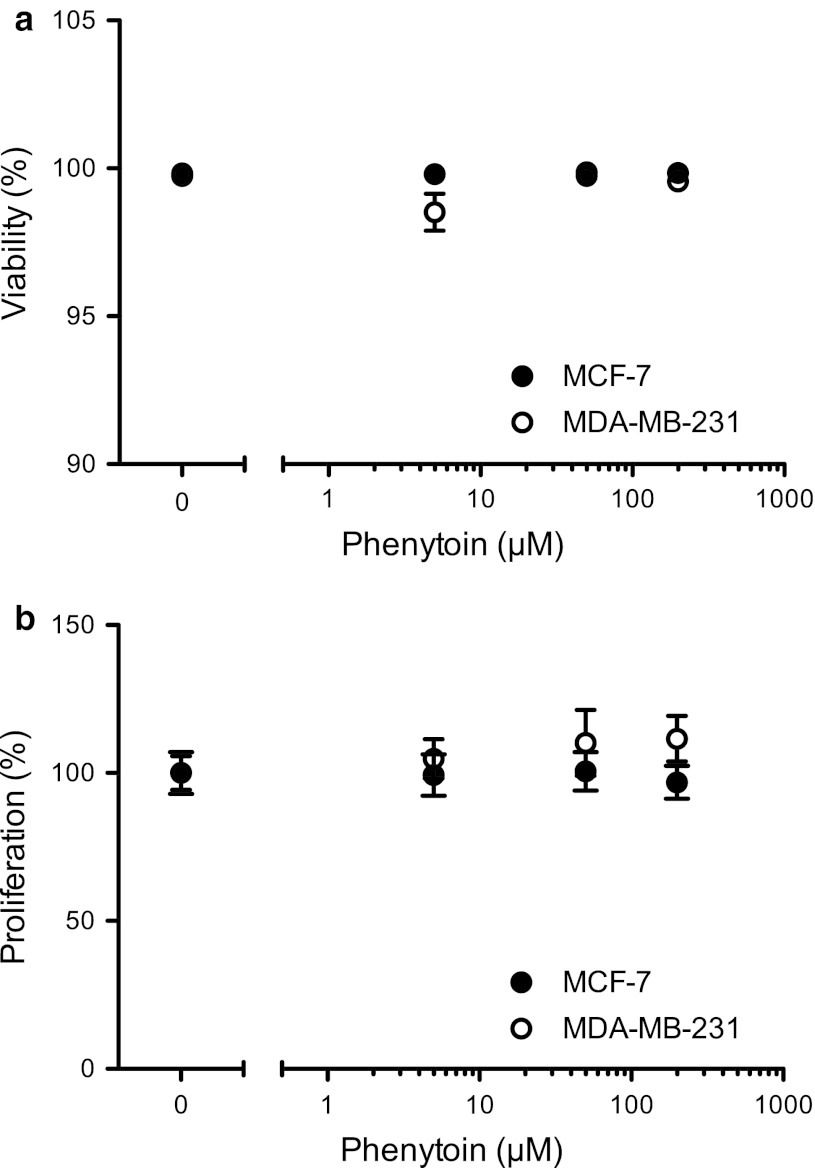
Effect of phenytoin on viability and proliferation. **a** Viability (%) of MCF-7 and MDA-MB-231 cells following treatment with phenytoin (5, 50, 200 μM) or vehicle for 24 h, normalized to control (*n* = 60). **b** Proliferation of MCF-7 and MDA-MB-231 cells following treatment with phenytoin (5, 50, 200 μM) or vehicle for 24 h, normalized to control (*n* ≥ 9). Data are mean ± SEM

The VGSC blocker TTX inhibits detachment, migration, galvanotaxis, and invasion of MDA-MB-231 cells [12–15]. TTX has no effect on these behaviors in cell lines that do not express VGSC-mediated Na^+^ currents, including MCF-7 cells [12, 13]. We therefore hypothesized that phenytoin would inhibit migration and invasion of MDA-MB-231 cells, but not MCF-7 cells. We first tested whether phenytoin was cytotoxic. Incubation with phenytoin (5–200 μM) for 24 h had no effect on viability of MCF-7 or MDA-MB-231 cells in a trypan blue exclusion assay (*P* = 0.93 and 0.67, respectively; Fig. 5a). Similarly, phenytoin had no effect on the proliferation of MCF-7 or MDA-MB-231 cells (*P* = 0.98 and 0.73, respectively; Fig. 5b).

**Fig. 6:**
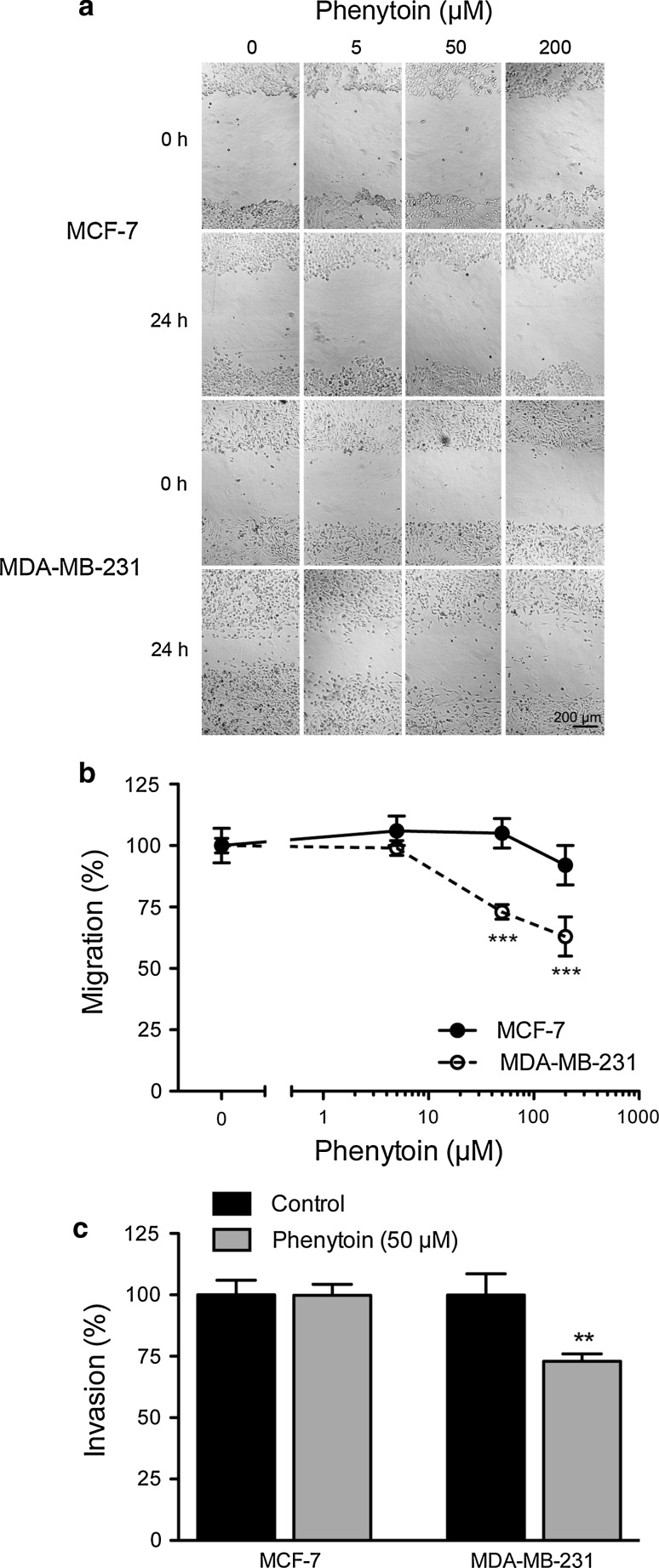
Effect of phenytoin on migration and invasion. **a** Representative images of MCF-7 and MDA-MB-231 cells in a wound healing assay at 0 h, and 24 h following treatment with phenytoin (5, 50, 200 μM) or vehicle. **b** Migration of MCF-7 and MDA-MB-231 cells treated with phenytoin (5, 50, 200 μM) or vehicle for 24 h in wound healing assay, normalized to control (*n* = 135 measurements per condition). **c** Invasion of MCF-7 and MDA-MB-231 cells ± phenytoin (50 μM) for 48 h, normalized to control (*n* = 9). Data are mean ± SEM. **P < 0.01; ***P < 0.001

We next tested the effect of phenytoin on migration in a wound heal assay. Phenytoin (5–200 μM; 24 h) had no effect on the migration of MCF-7 cells (*P* = 0.41), but significantly reduced the migration of MDA-MB-231 cells by 27.3 ± 1.9 % at 50 μM, and 37.2 ± 1.8 % at 200 μM (*P* < 0.001; Fig. 6a, b). Similarly, phenytoin (50 μM; 48 h) had no effect on the invasion of MCF-7 cells (*P* = 0.99), but significantly reduced the invasion of MDA-MB-231 cells by 27.1 ± 3.1 % (*P* < 0.05; Fig. 6c). In conclusion, phenytoin significantly inhibited the migration and invasion of MDA-MB-231 cells, which express functional VGSCs.

## Discussion

The expression of VGSCs in electrically excitable cells, and their importance as therapeutic targets in excitability-related disorders, e.g., epilepsy, has been long established [19]. However, it is only more recently that their importance in cancer has begun to be identified [8]. *SCN5A*/Na_v_1.5 is expressed, predominantly in its neonatal splice form, in MDA-MB-231 cells, which are triple negative for ER/PR/HER2 [12]. Neonatal Na_v_1.5 is expressed in cells of epithelial origin in BCa biopsies, but is absent in normal breast [12]. We found several studies in Oncomine in which *SCN5A* was up-regulated in BCa compared with normal tissue. However, there was no relationship between *SCN5A* expression and ER/PR/HER2 status in these datasets. *SCN5A* expression has been reported in other cancers, including lymphoma [51], neuroblastoma [52], colorectal [53], lung [24], and ovarian cancers [54]. In addition, other VGSC subtypes have been reported in melanoma [55], mesothelioma [56], cervical [57, 58], lung [24], ovarian [54], and prostate cancers [59, 60].

*SCN5A* was more highly expressed in samples from patients who had a recurrence, metastasis, or died within 5 years. *SCN5A* expression as an outlier also associated with metastasis. These findings agree with a previous report correlating neonatal *SCN5A* variant mRNA in BCa biopsies with lymph node metastasis [12]. The microarray probes used in the studies in Oncomine do not distinguish between neonatal and adult *SCN5A* splice variants. However, the neonatal splice variant is predominant in MDA-MB-231 cells and in BCa biopsies in which both variants were studied [12, 15]. In conclusion, our data support the notion that *SCN5A* is up-regulated in BCa, and may be a marker for poor prognosis.

Blocking Na^+^ current carried by Na_v_1.5 in MDA-MB-231 cells with TTX, siRNA, or antibodies, inhibits migration, galvanotaxis, and invasion, and enhances adhesion [12–15]. Similarly, blocking Na^+^ current suppresses these behaviors in cell lines from other cancers (reviewed in [8]). VGSCs are expressed at lamellipodia of MDA-MB-231 and MCF-7 cells, consistent with VGSCs functioning within these regions to regulate adhesion, migration, and invasion. Importantly, the lamellipodial/cell body α subunit expression ratio is higher in MDA-MB-231 cells than MCF-7 cells, consistent with α subunits regulating migration and invasion in the former, rather than the latter cell line. It is possible that MCF-7 cells also express other VGSC α subunit variants that have impaired conduction [48]. Thus, in addition to being a marker for metastatic BCa, Na_v_1.5 may be a useful therapeutic target for slowing disease progression and/or metastasis.

A number of VGSC-targeting drugs are used to treat other diseases, e.g., epilepsy [19]. Several of these drugs, including phenytoin, bind preferentially to VGSCs that are inactivated [20, 50]. VGSCs typically inactivate within a few milliseconds of opening following depolarization, and remain in that state until the membrane repolarizes [5]. Several subtypes, including Na_v_1.5, do not inactivate completely, and continue to carry a small steady-state persistent Na^+^ current at depolarized potentials [61, 62]. Cancer cells typically have a more depolarized membrane potential than normal epithelial cells, or terminally differentiated excitable cells, e.g., neurons [63]. Thus, it is the persistent component of Na^+^ current that is likely to be predominant in BCa cells, potentiating invasion and migration. Phenytoin significantly inhibited both transient and persistent Na^+^ currents in MDA-MB-231 cells. Importantly, the tonic block was greater at more depolarized holding voltages, suggesting that phenytoin may be a highly effective VGSC blocker in depolarized cancer cells. This is the first report of phenytoin inhibiting Na^+^ current in cancer cells, and agrees with the effect of this drug on VGSCs in other cells, e.g., [20, 50].

Our electrophysiological data suggest that phenytoin may be a useful therapeutic agent for blocking Na^+^ current in BCa cells. Na^+^ current enhances invasion by promoting cysteine cathepsin activity in caveolae [16, 17], and Na_v_1.5 has been proposed to be a key regulator of invasion-controlling genes [53]. We found that phenytoin significantly inhibited migration and invasion in MDA-MB-231 cells expressing Na^+^ currents by ~30 %. This is equivalent to the effect of blocking VGSCs using TTX, siRNA, or antibodies, reported previously [12, 13, 15]. Phenytoin had no effect on the migration or invasion of MCF-7 cells, which do not express Na^+^ currents. Phenytoin also had no effect on the proliferation of either cell line, consistent with previous reports indicating that VGSCs regulate cell migration and invasion, but not proliferation [8]. Phenytoin has been shown to inhibit HERG channels at significantly higher concentrations (IC_50_ for HERG > 300 μM, vs. IC_50_ for VGSC ~10 μM) [19, 21]. MCF-7 cells express outward K^+^ currents, although the channel has not yet been identified [12, 17]. In contrast, MDA-MB-231 cells do not express any voltage-dependent K^+^ (e.g., HERG) currents [12, 17]. Together, these data suggest that phenytoin (50, 200 μM) inhibited VGSC-dependent migration and invasion in MDA-MB-231 cells by suppressing Na^+^ current, rather than inhibiting another target, e.g., HERG channels.

The concentration of phenytoin that inhibits Na^+^ current, migration, and invasion (50 μM) is within the serum therapeutic range used in clinical settings for treatment of epilepsy [49]. Our data suggest that repurposing phenytoin to BCa warrants further study as a potential new treatment. However, it is possible that the effect of phenytoin on BCa in vivo may be more complex, given that VGSCs are expressed on a multitude of cell types, and this would require further investigation [8, 64, 65]. Another FDA-approved VGSC-blocking drug, riluzole, which also inhibits metabotropic glutamate receptors, has shown promise in treating melanomas, and reduces BCa tumor volume in mice [66, 67]. Use of VGSC-blocking local anesthetics during radical prostatectomy surgery is associated with substantially reduced recurrence and metastasis [68]. In conclusion, a growing body of evidence supports the notion that VGSCs may be useful therapeutic targets in cancer.

Our data support the hypothesis that *SCN5A* is up-regulated in BCa, and plays a role in metastasis. In agreement with previous reports [12, 53], *SCN5A* expression may be an important event in progression toward metastasis. Together with other studies [12, 13, 15, 53], this study suggests that Na_v_1.5-mediated Na^+^ current favors an invasive phenotype. We therefore propose that using VGSC-blocking drugs, in particular those that target persistent Na^+^ current, should be considered for further study as a potential strategy to improve patient outcomes in metastatic BCa.

## Abbreviations

AUC: Area under curve
BCa: Breast cancer
CAM: Cell adhesion molecule
COPA: Cancer outlier profile analysis
DAPI: 4′,6-diamidino-2-phenylindole
DCIS: Ductal carcinoma in situ
ER: Estrogen receptor
HER2: Human epidermal growth factor receptor 2
HERG: Human *Ether*-*à*-*go*-*go*-related gene
IBCa: Invasive breast cancer
IDBCa: Invasive ductal breast carcinoma
IL-6: Interleukin-6
IMBCa: Invasive mixed breast carcinoma
*k*: Slope factor
MI: Motility index
MTT: 3-[4,5-dimethylthiazol-2-yl]-2,5-diphenyltetrazolium bromide
PR: Progesterone receptor
PSA: Prostate-specific antigen
ROC: Receiver operating characteristic
SEM: Standard error of the mean
TCGA: The Cancer Genome Atlas
TTX: Tetrodotoxin
VGSC: Voltage-gated Na^+^ channel
*V*_m_: Membrane potential
*V*_1/2_: Half inactivation voltage

## Footnotes

1
http://tcga-data.nci.nih.gov/tcga/.

## References

[1] Jemal A, Bray F, Center MM, Ferlay J, Ward E, Forman D (2011). Global cancer statistics. CA Cancer J Clin.

[2] Rugo HS (2008). The importance of distant metastases in hormone-sensitive breast cancer. Breast.

[3] Suva LJ, Griffin RJ, Makhoul I (2009). Mechanisms of bone metastases of breast cancer. Endocr Relat Cancer.

[4] Catterall WA (2000). From ionic currents to molecular mechanisms: the structure and function of voltage-gated sodium channels. Neuron.

[5] Hille B (1992). Ionic channels of excitable membranes.

[6] Brackenbury WJ, Isom LL (2008). Voltage-gated Na^+^ channels: potential for beta subunits as therapeutic targets. Expert Opin Ther Targets.

[7] Isom LL, Catterall WA (1996). Na^+^ channel subunits and Ig domains. Nature.

[8] Brackenbury WJ, Djamgoz MB, Isom LL (2008). An emerging role for voltage-gated Na^+^ channels in cellular migration: regulation of central nervous system development and potentiation of invasive cancers. Neuroscientist.

[9] Brackenbury WJ, Calhoun JD, Chen C, Miyazaki H, Nukina N, Oyama F, Ranscht B, Isom LL (2010). Functional reciprocity between Na^+^ channel Nav1.6 and β1 subunits in the coordinated regulation of excitability and neurite outgrowth. Proc Natl Acad Sci USA.

[10] Brackenbury WJ, Davis TH, Chen C, Slat EA, Detrow MJ, Dickendesher TL, Ranscht B, Isom LL (2008). Voltage-gated Na^+^ channel β1 subunit-mediated neurite outgrowth requires fyn kinase and contributes to central nervous system development in vivo. J Neurosci.

[11] Brackenbury WJ, Isom LL (2011). Na^+^ channel β subunits: overachievers of the ion channel family. Front Pharmacol.

[12] Fraser SP, Diss JK, Chioni AM, Mycielska M, Pan H, Yamaci RF, Pani F, Siwy Z, Krasowska M, Grzywna Z, Brackenbury WJ, Theodorou D, Koyuturk M, Kaya H, Battaloglu E, Tamburo De Bella M, Slade MJ, Tolhurst R, Palmieri C, Jiang J, Latchman DS, Coombes RC, Djamgoz MB (2005). Voltage-gated sodium channel expression and potentiation of human breast cancer metastasis. Clin Cancer Res.

[13] Roger S, Besson P, Le Guennec JY (2003). Involvement of a novel fast inward sodium current in the invasion capacity of a breast cancer cell line. Biochim Biophys Acta.

[14] Palmer CP, Mycielska ME, Burcu H, Osman K, Collins T, Beckerman R, Perrett R, Johnson H, Aydar E, Djamgoz MB (2008). Single cell adhesion measuring apparatus (SCAMA): application to cancer cell lines of different metastatic potential and voltage-gated Na^+^ channel expression. Eur Biophys J.

[15] Brackenbury WJ, Chioni AM, Diss JK, Djamgoz MB (2007). The neonatal splice variant of Na_v_1.5 potentiates in vitro metastatic behaviour of MDA-MB-231 human breast cancer cells. Breast Cancer Res Treat.

[16] Gillet L, Roger S, Besson P, Lecaille F, Gore J, Bougnoux P, Lalmanach G, Le Guennec JY (2009). Voltage-gated sodium channel activity promotes cysteine cathepsin-dependent invasiveness and colony growth of human cancer cells. J Biol Chem.

[17] Brisson L, Gillet L, Calaghan S, Besson P, Le Guennec JY, Roger S, Gore J (2011). Na(V)1.5 enhances breast cancer cell invasiveness by increasing NHE1-dependent H(+) efflux in caveolae. Oncogene.

[18] Chioni AM, Brackenbury WJ, Calhoun JD, Isom LL, Djamgoz MB (2009). A novel adhesion molecule in human breast cancer cells: voltage-gated Na^+^ channel β1 subunit. Int J Biochem Cell Biol.

[19] Mantegazza M, Curia G, Biagini G, Ragsdale DS, Avoli M (2010). Voltage-gated sodium channels as therapeutic targets in epilepsy and other neurological disorders. Lancet Neurol.

[20] Ragsdale DS, Scheuer T, Catterall WA (1991). Frequency and voltage-dependent inhibition of type IIA Na^+^ channels, expressed in a mammalian cell line, by local anesthetic, antiarrhythmic, and anticonvulsant drugs. Mol Pharmacol.

[21] Danielsson BR, Lansdell K, Patmore L, Tomson T (2003). Phenytoin and phenobarbital inhibit human HERG potassium channels. Epilepsy Res.

[22] Abdul M, Hoosein N (2001). Inhibition by anticonvulsants of prostate-specific antigen and interleukin-6 secretion by human prostate cancer cells. Anticancer Res.

[23] Fraser SP, Salvador V, Manning EA, Mizal J, Altun S, Raza M, Berridge RJ, Djamgoz MB (2003). Contribution of functional voltage-gated Na^+^ channel expression to cell behaviors involved in the metastatic cascade in rat prostate cancer: I. lateral motility. J Cell Physiol.

[24] Onganer PU, Djamgoz MB (2005). Small-cell lung cancer (human): potentiation of endocytic membrane activity by voltage-gated Na^+^ channel expression in vitro. J Membr Biol.

[25] Rhodes DR, Yu J, Shanker K, Deshpande N, Varambally R, Ghosh D, Barrette T, Pandey A, Chinnaiyan AM (2004). ONCOMINE: a cancer microarray database and integrated data-mining platform. Neoplasia.

[26] Cao Q, Gery S, Dashti A, Yin D, Zhou Y, Gu J, Koeffler HP (2009). A role for the clock gene per1 in prostate cancer. Cancer Res.

[27] Crea F, Hurt EM, Farrar WL (2010). Clinical significance of Polycomb gene expression in brain tumors. Mol Cancer.

[28] Wurmbach E, Yuen T, Sealfon SC (2003). Focused microarray analysis. Methods.

[29] Wurmbach E, Yuen T, Ebersole BJ, Sealfon SC (2001). Gonadotropin-releasing hormone receptor-coupled gene network organization. J Biol Chem.

[30] Morey JS, Ryan JC, Van Dolah FM (2006). Microarray validation: factors influencing correlation between oligonucleotide microarrays and real-time PCR. Biol Proced Online.

[31] Tomlins SA, Rhodes DR, Perner S, Dhanasekaran SM, Mehra R, Sun XW, Varambally S, Cao X, Tchinda J, Kuefer R, Lee C, Montie JE, Shah RB, Pienta KJ, Rubin MA, Chinnaiyan AM (2005). Recurrent fusion of TMPRSS2 and ETS transcription factor genes in prostate cancer. Science.

[32] McShane LM, Altman DG, Sauerbrei W, Taube SE, Gion M, Clark GM (2006). REporting recommendations for tumor MARKer prognostic studies (REMARK). Breast Cancer Res Treat.

[33] Uphoff CC, Gignac SM, Drexler HG (1992). Mycoplasma contamination in human leukemia cell lines. I. Comparison of various detection methods. J Immunol Methods.

[34] Masters JR, Thomson JA, Daly-Burns B, Reid YA, Dirks WG, Packer P, Toji LH, Ohno T, Tanabe H, Arlett CF, Kelland LR, Harrison M, Virmani A, Ward TH, Ayres KL, Debenham PG (2001). Short tandem repeat profiling provides an international reference standard for human cell lines. Proc Natl Acad Sci USA.

[35] Wong HK, Sakurai T, Oyama F, Kaneko K, Wada K, Miyazaki H, Kurosawa M, De Strooper B, Saftig P, Nukina N (2005). Beta subunits of voltage-gated sodium channels are novel substrates of BACE1 and gamma-secretase. J Biol Chem.

[36] Brackenbury WJ, Djamgoz MB (2006). Activity-dependent regulation of voltage-gated Na^+^ channel expression in Mat-LyLu rat prostate cancer cell line. J Physiol.

[37] Hu J, Mukhopadhyay A, Craig AW (2011). Transducer of Cdc42-dependent actin assembly promotes epidermal growth factor-induced cell motility and invasiveness. J Biol Chem.

[38] Armstrong CM, Bezanilla F (1977). Inactivation of the sodium channel. II. Gating current experiments. J Gen Physiol.

[39] Fraser SP, Ding Y, Liu A, Foster CS, Djamgoz MB (1999). Tetrodotoxin suppresses morphological enhancement of the metastatic MAT-LyLu rat prostate cancer cell line. Cell Tissue Res.

[40] Grimes JA, Fraser SP, Stephens GJ, Downing JE, Laniado ME, Foster CS, Abel PD, Djamgoz MB (1995). Differential expression of voltage-activated Na^+^ currents in two prostatic tumour cell lines: contribution to invasiveness in vitro. FEBS Lett.

[41] Finak G, Bertos N, Pepin F, Sadekova S, Souleimanova M, Zhao H, Chen H, Omeroglu G, Meterissian S, Omeroglu A, Hallett M, Park M (2008). Stromal gene expression predicts clinical outcome in breast cancer. Nat Med.

[42] Radvanyi L, Singh-Sandhu D, Gallichan S, Lovitt C, Pedyczak A, Mallo G, Gish K, Kwok K, Hanna W, Zubovits J, Armes J, Venter D, Hakimi J, Shortreed J, Donovan M, Parrington M, Dunn P, Oomen R, Tartaglia J, Berinstein NL (2005). The gene associated with trichorhinophalangeal syndrome in humans is overexpressed in breast cancer. Proc Natl Acad Sci USA.

[43] Schmidt M, Bohm D, von Torne C, Steiner E, Puhl A, Pilch H, Lehr HA, Hengstler JG, Kolbl H, Gehrmann M (2008). The humoral immune system has a key prognostic impact in node-negative breast cancer. Cancer Res.

[44] Desmedt C, Piette F, Loi S, Wang Y, Lallemand F, Haibe-Kains B, Viale G, Delorenzi M, Zhang Y, d’Assignies MS, Bergh J, Lidereau R, Ellis P, Harris AL, Klijn JG, Foekens JA, Cardoso F, Piccart MJ, Buyse M, Sotiriou C (2007). Strong time dependence of the 76-gene prognostic signature for node-negative breast cancer patients in the TRANSBIG multicenter independent validation series. Clin Cancer Res.

[45] Boersma BJ, Reimers M, Yi M, Ludwig JA, Luke BT, Stephens RM, Yfantis HG, Lee DH, Weinstein JN, Ambs S (2008). A stromal gene signature associated with inflammatory breast cancer. Int J Cancer.

[46] Shah BS, Rush AM, Liu S, Tyrrell L, Black JA, Dib-Hajj SD, Waxman SG (2004). Contactin associates with sodium channel Na_v_1.3 in native tissues and increases channel density at the cell surface. J Neurosci.

[47] Lopez-Santiago LF, Brackenbury WJ, Chen C, Isom LL (2011). Na^+^ channel Scn1b gene regulates dorsal root ganglion nociceptor excitability in vivo. J Biol Chem.

[48] Wilde AA, Brugada R (2011). Phenotypical manifestations of mutations in the genes encoding subunits of the cardiac sodium channel. Circ Res.

[49] Turnbull DM, Rawlins MD, Weightman D, Chadwick DW (1984). “Therapeutic” serum concentration of phenytoin: the influence of seizure type. J Neurol Neurosurg Psychiatry.

[50] Lenkowski PW, Ko SH, Anderson JD, Brown ML, Patel MK (2004). Block of human Na_v_1.5 sodium channels by novel alpha-hydroxyphenylamide analogues of phenytoin. Eur J Pharm Sci.

[51] Fraser SP, Diss JK, Lloyd LJ, Pani F, Chioni AM, George AJ, Djamgoz MB (2004). T-lymphocyte invasiveness: control by voltage-gated Na^+^ channel activity. FEBS Lett.

[52] Ou SW, Kameyama A, Hao LY, Horiuchi M, Minobe E, Wang WY, Makita N, Kameyama M (2005). Tetrodotoxin-resistant Na^+^ channels in human neuroblastoma cells are encoded by new variants of Na_v_1.5/SCN5A. Eur J Neurosci.

[53] House CD, Vaske CJ, Schwartz A, Obias V, Frank B, Luu T, Sarvazyan N, Irby RB, Strausberg RL, Hales T, Stuart J, Lee NH (2010). Voltage-gated Na^+^ channel SCN5A is a key regulator of a gene transcriptional network that controls colon cancer invasion. Cancer Res.

[54] Gao R, Shen Y, Cai J, Lei M, Wang Z (2010). Expression of voltage-gated sodium channel alpha subunit in human ovarian cancer. Oncol Rep.

[55] Carrithers MD, Chatterjee G, Carrithers LM, Offoha R, Iheagwara U, Rahner C, Graham M, Waxman SG (2009). Regulation of podosome formation in macrophages by a novel splice variant of the sodium channel SCN8A. J Biol Chem.

[56] Fulgenzi G, Graciotti L, Faronato M, Soldovieri MV, Miceli F, Amoroso S, Annunziato L, Procopio A, Taglialatela M (2006). Human neoplastic mesothelial cells express voltage-gated sodium channels involved in cell motility. Int J Biochem Cell Biol.

[57] Diaz D, Delgadillo D, Hernandez-Gallegoz E, Ramirez-Dominguez M, Hinojosa L, Ortiz C, Berumen J, Camacho J, Gomora J (2007). Functional expression of voltage-gated sodium channels in primary cultures of human cervical cancer. J Cell Physiol.

[58] Hernandez-Plata E, Ortiz CS, Marquina-Castillo B, Medina-Martinez I, Alfaro A, Berumen J, Rivera M, Gomora JC (2012) Overexpression of Na(V) 1.6 channels is associated with the invasion capacity of human cervical cancer. Int J Cancer 130:2013–2023 10.1002/ijc.26210 21630263

[59] Diss JK, Archer SN, Hirano J, Fraser SP, Djamgoz MB (2001). Expression profiles of voltage-gated Na^+^ channel alpha-subunit genes in rat and human prostate cancer cell lines. Prostate.

[60] Diss JK, Fraser SP, Walker MM, Patel A, Latchman DS, Djamgoz MB (2008). Beta-subunits of voltage-gated sodium channels in human prostate cancer: quantitative in vitro and in vivo analyses of mRNA expression. Prostate Cancer Prostatic Dis.

[61] Crill WE (1996). Persistent sodium current in mammalian central neurons. Annu Rev Physiol.

[62] Ju YK, Saint DA, Gage PW (1996). Hypoxia increases persistent sodium current in rat ventricular myocytes. J Physiol.

[63] Kunzelmann K (2005). Ion channels and cancer. J Membr Biol.

[64] Verrotti A, D’Egidio C, Mohn A, Coppola G, Parisi P, Chiarelli F (2011). Antiepileptic drugs, sex hormones, and PCOS. Epilepsia.

[65] Beghi E, Shorvon S (2011). Antiepileptic drugs and the immune system. Epilepsia.

[66] Speyer CL, Smith JS, Banda M, Devries JA, Mekani T, Gorski DH (2012) Metabotropic glutamate receptor-1: a potential therapeutic target for the treatment of breast cancer. Breast Cancer Res Treat 132(2):565–573 10.1007/s10549-011-1624-x PMC3898178 21681448

[67] Yip D, Le MN, Chan JL, Lee JH, Mehnert JA, Yudd A, Kempf J, Shih WJ, Chen S, Goydos JS (2009). A phase 0 trial of riluzole in patients with resectable stage III and IV melanoma. Clin Cancer Res.

[68] Biki B, Mascha E, Moriarty DC, Fitzpatrick JM, Sessler DI, Buggy DJ (2008). Anesthetic technique for radical prostatectomy surgery affects cancer recurrence: a retrospective analysis. Anesthesiology.

[69] West M, Blanchette C, Dressman H, Huang E, Ishida S, Spang R, Zuzan H, Olson JA, Marks JR, Nevins JR (2001). Predicting the clinical status of human breast cancer by using gene expression profiles. Proc Natl Acad Sci USA.

[70] van de Vijver MJ, He YD, van’t Veer LJ, Dai H, Hart AA, Voskuil DW, Schreiber GJ, Peterse JL, Roberts C, Marton MJ, Parrish M, Atsma D, Witteveen A, Glas A, Delahaye L, van der Velde T, Bartelink H, Rodenhuis S, Rutgers ET, Friend SH, Bernards R (2002). A gene-expression signature as a predictor of survival in breast cancer. N Engl J Med.

[71] Bild AH, Yao G, Chang JT, Wang Q, Potti A, Chasse D, Joshi MB, Harpole D, Lancaster JM, Berchuck A, Olson JA, Marks JR, Dressman HK, West M, Nevins JR (2006). Oncogenic pathway signatures in human cancers as a guide to targeted therapies. Nature.

[72] Julka PK, Chacko RT, Nag S, Parshad R, Nair A, Oh DS, Hu Z, Koppiker CB, Nair S, Dawar R, Dhindsa N, Miller ID, Ma D, Lin B, Awasthy B, Perou CM (2008). A phase II study of sequential neoadjuvant gemcitabine plus doxorubicin followed by gemcitabine plus cisplatin in patients with operable breast cancer: prediction of response using molecular profiling. Br J Cancer.

[73] Waddell N, Cocciardi S, Johnson J, Healey S, Marsh A, Riley J, da Silva L, Vargas AC, Reid L, Simpson PT, Lakhani SR, Chenevix-Trench G (2010). Gene expression profiling of formalin-fixed, paraffin-embedded familial breast tumours using the whole genome-DASL assay. J Pathol.

